# Inhibition of Wnt signalling by Notch via two distinct mechanisms

**DOI:** 10.1038/s41598-021-88618-5

**Published:** 2021-04-27

**Authors:** Ahmet Acar, Ana Hidalgo-Sastre, Michael K. Leverentz, Christopher G. Mills, Simon Woodcock, Martin Baron, Giovanna M. Collu, Keith Brennan

**Affiliations:** 1 Department of Biological Sciences, Middle East Technical University, Universiteler Mah. Dumlupınar Bulvarı 1, 06800 Çankaya, Ankara, Turkey; 2 Faculty of Biology, Medicine and Health, Manchester Academic Health Science Centre, University of Manchester, Oxford Road, Manchester, M13 9PT UK; 3 Department of Developmental and Regenerative Biology, Icahn School of Medicine At Mount Sinai, One Gustave L. Levy Place, Box 1020, New York, NY 10029 USA

**Keywords:** Cell biology, Developmental biology

## Abstract

Notch and Wnt are two essential signalling pathways that help to shape animals during development and to sustain adult tissue homeostasis. Although they are often active at the same time within a tissue, they typically have opposing effects on cell fate decisions. In fact, crosstalk between the two pathways is important in generating the great diversity of cell types that we find in metazoans. Several different mechanisms have been proposed that allow Notch to limit Wnt signalling, driving a Notch-ON/Wnt-OFF state. Here we explore these different mechanisms in human cells and demonstrate two distinct mechanisms by which Notch itself, can limit the transcriptional activity of β-catenin. At the membrane, independently of DSL ligands, Notch1 can antagonise β-catenin activity through an endocytic mechanism that requires its interaction with Deltex and sequesters β-catenin into the membrane fraction. Within the nucleus, the intracellular domain of Notch1 can also limit β-catenin induced transcription through the formation of a complex that requires its interaction with RBPjκ. We believe these mechanisms contribute to the robustness of cell-fate decisions by sharpening the distinction between opposing Notch/Wnt responses.

## Introduction

Animal development and adult tissue renewal are controlled by a handful of different signalling pathways that define a bewildering array of different cell types^1^. This clearly raises the question of how so few signalling pathways can generate such diversity. The answer lies, in part, in crosstalk between the signalling pathways that allows one pathway to regulate or alter the output of another. The Notch and Wnt signalling pathways are a good example of this^2^. These pathways are often active at the same time to regulate the development and maintenance of a particular tissue but frequently have opposing effects on cell fate specification^3^. Therefore, it is not surprising that signalling through the two pathways is under strict control to prevent a conflict between them and to resolve into either Wnt-ON/Notch-OFF or Notch-ON/Wnt-OFF. This is particularly important to allow the progression of a cell along a particular lineage where subsequent steps are controlled by Wnt and Notch signalling. For example, Notch signalling promotes the differentiation of stem cells in both the skin and the mammary gland by inhibiting the Wnt signal that supports the self-renewal of these stem cells^4^. Similarly, resolving competition between the two pathways is required for the successful differentiation of enterocytes and secretory cells within the intestinal epithelium^5–8^.

It is well established that the key parameter of the Wnt signalling pathway is the stability and localisation of the soluble pool of β-catenin^9–12^. In the absence of Wnt ligand, the free cytosolic β-catenin interacts with a destruction complex, comprising CK1, APC, Axin and GSK3β, which phosphorylates β-catenin to target it for degradation via the proteosome. The binding of Wnt ligand to the cell surface receptors Frizzled and LRP recruits the cytoplasmic adaptor protein Dishevelled and the destruction complex to the membrane. Consequently, β-catenin is no longer phosphorylated and accumulates in the cytosol and nucleus. Within the nucleus, β-catenin induces the expression of downstream target genes by interacting with members of the TCF/LEF family of DNA binding proteins. Notch signalling is triggered by the interaction of Notch receptors and DSL (Delta, Serrate, Lag2) ligands on adjacent cells^13^. This leads to the proteolytic cleavage of Notch to release the intracellular domain (NICD), which translocates to the nucleus to induce target gene transcription in a complex with the DNA binding protein RBPj and the transcriptional co-activator Mastermind-like (MAML).

Over recent years, several mechanisms have been proposed to explain the crosstalk between the Notch and Wnt pathways that allow signalling through the two pathways to be resolved into Notch-ON/Wnt-OFF. Studies in Drosophila and mammalian stem and cancer cells have suggested that Notch present within the plasma membrane can modulate the amount and transcriptional activity of Armadillo/β-catenin^14–16^ by associating with Armadillo/β-catenin present at the membrane and promoting its degradation through endosomal trafficking. In contrast, several other studies in Xenopus, mice and cell lines have suggested that the inhibition of Wnt signalling is mediated by the nuclear form of Notch, NICD, and may require the expression of downstream targets^14,17–19^. However, it is difficult to know from these studies whether one or both proposed mechanisms are required for Notch to affect the crosstalk on β-catenin, as many of the experiments have been completed with forms of the Notch protein that can localise to the membrane upon synthesis and the nucleus after γ-secretase mediated cleavage.

Here, we demonstrate that both a signalling inactive form of Notch that is restricted to the membrane fraction and a nuclear form of Notch, NICD, can inhibit Wnt/β-catenin signalling, although there is a mark difference in their potency. The membrane-restricted form of Notch sequesters β-catenin to the membrane fraction preventing its interaction with TCF/LEF and requires endocytosis to inhibit Wnt signalling. The nuclear form of Notch can also disrupt Wnt/β-catenin signalling by forming a complex with β-catenin, but does not require the transcription of downstream Notch target genes. Interestingly, we find that the interaction between β-catenin and nuclear Notch is stabilised by RBPj, although RBPj cannot form a complex with β-catenin itself. This may explain the marked difference in potency between the two different forms of Notch, as RBPj is nuclear restricted. Together, our results indicate that the interaction between Notch and β-catenin can limit Wnt signalling to establish a Notch-ON/Wnt-OFF state to allow robust cell-fate decisions during embryonic development and tissue homeostasis.

## Results

### Notch modulates the transcriptional activity of β-catenin

**Figure 1:**
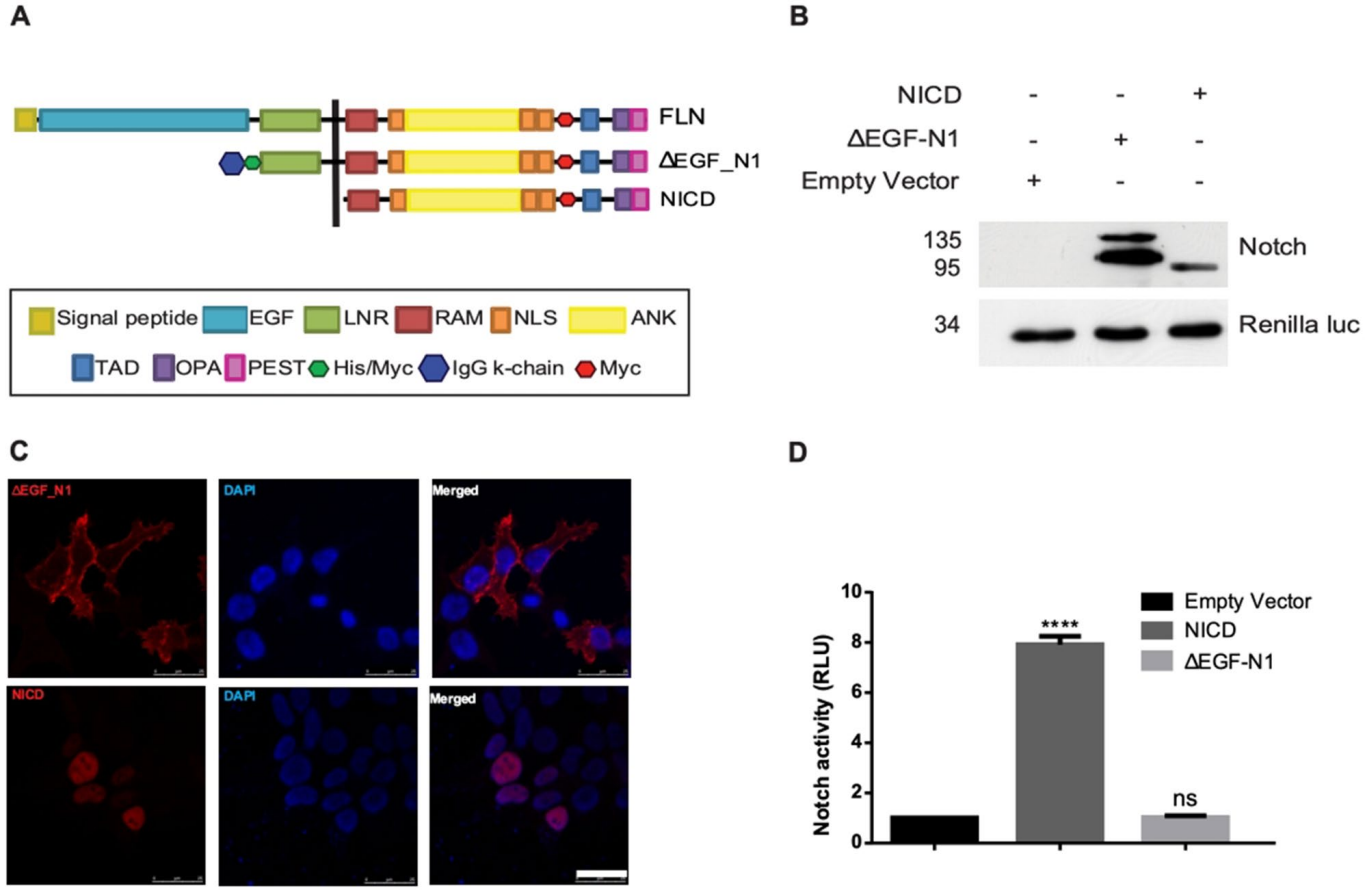
Characterization of ΔEGF_N1 and NICD constructs. (**A**) Schematic showing the structure of the full length, ΔEGF_N1 and NICD proteins. (**B**) Western blot analysis of ΔEGF_N1, NICD constructs and empty vector. Expressed proteins were detected by probing the western blot with an antibody that recognises the myc epitope tag found within both proteins. Renilla luciferase is shown as a loading control. The position of molecular weight markers is shown in KDa. (**C**) Immunofluorescence analysis of ΔEGF_N1 and NICD proteins. Scale bar is 25 μm. (**D**) Unlike NICD, ΔEGF_N1 failed to activate Notch signalling. HEK293T cells were transfected with p10xRbpj-luc either alone or with plasmids encoding the ΔEGF_N1 and NICD proteins. Experiments were performed in triplicate. pRL-CMV was used as a transfection control and cells were lysed 48 h post transfection to determine luciferase activity. Data are presented as mean fold change (± SEM) in RLU (NS *P* > 0.05; ****P < 0.0001 one-way ANOVA and Tukey’s post-hoc test, N = 3). Original uncropped Western Blot images of panel B are shown in Supplementary Fig. 5

**Figure 2:**
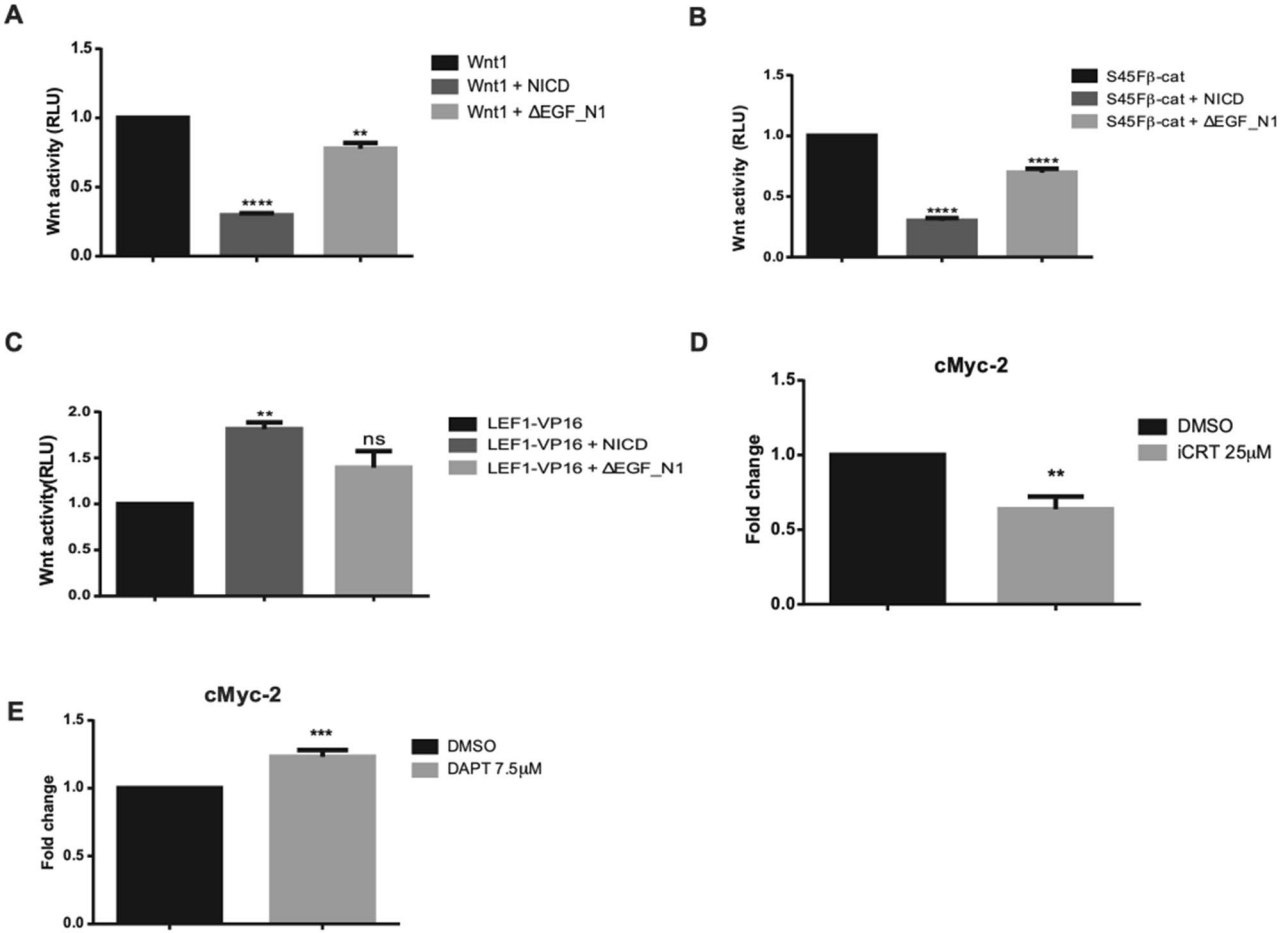
Both the ΔEGF_N1 and NICD protein can inhibit Wnt signalling. (**A**,**B**) ΔEGF_N1 and NICD inhibited Wnt1 and S45Fβ-catenin-driven Wnt signalling, whereas (**C**) neither could inhibit LEF1-VP16 induced transcription. HEK293T cells were transfected with Wnt reporter plasmid pTCFAdTATA (**A**,**B**, **C**) and Wnt signalling was activated by expressing Wnt1 (**A**), an active form of β-catenin (S45Fβ-catenin) (**B**), or LEF1-VP16 (**C**) alone or in the presence of the ΔEGF_N1 or NICD proteins as indicated. (**D**,**E**) Quantitative PCR (qPCR) analysis of *c-Myc* gene in HCT116 cells. As expected, *c-Myc* expression was reduced when Wnt signaling was blocked with the Wnt inhibitor iCRT (**D**). Inhibiting Notch signalling with DAPT treatment increased *c-Myc* expression, indicating endogenous Notch signalling is suppressing *c-Myc* expression (**E**). Luciferase assays were undertaken in HEK293T cells and were performed in triplicate. pRL-CMV was used as a transfection control and cells were lysed 48 h post transfection to determine luciferase activity. Data are presented as mean fold change (± SEM) in RLU or as mean fold change in gene expression (NS *P* > 0.05; **P < 0.01, ***P < 0.001, ****P < 0.0001 one-way ANOVA and Tukey’s post-hoc test, N = 3).

To determine whether both a membrane localised and a nuclear form of Notch can inhibit Wnt signalling, we initially generated plasmids expressing two distinct Notch proteins, ΔEGF_N1 and NICD. The ΔEGF_N1 construct lacked the 36 extracellular EGF-like repeats, preventing ligand binding, but retained the LNR domain that masks the S2 cleavage site (Fig. 1A). As expected, ΔEGF_N1 protein underwent S1 cleavage which occurs during protein maturation (Fig. 1B), was found within the membrane fraction (Fig. 1C), and failed to induce the expression of an RBPj-dependent reporter gene (Fig. 1D). In contrast, the NICD construct, which comprises the intracellular domain only (Fig. 1A), was expressed as expectedly (Fig. 1B), found exclusively within the nucleus (Fig. 1C) and robustly activated RBPj-dependent transcription (Fig. 1D). Expressing either of these proteins with the Wnt1 protein by co-transfection reduced the response of HEK293T cells to Wnt1 (Fig. 2A); Wnt signalling was monitored in the HEK293T cells by co-transfecting them with the pTCFAdTATA reporter plasmid, which contains a minimal adenoviral TATA box regulated by four TCF/LEF binding sites and is more strongly activated by the Wnt pathway than the TOPflash reporter plasmid (Fig. S1A,B). There was, however, a marked difference in the potency of the two Notch proteins with NICD reducing Wnt signalling more markedly. To establish where in the Wnt pathway the two Notch proteins were regulating signalling, we activated the Wnt pathway at different points by expressing S45Fβ-catenin (a stabilised form of β-catenin;^20^) or LEF1-VP16 (a constitutively active form of the LEF1 transcription factor;^21^). ΔEGF_N1 and NICD were both able to inhibit S45Fβ-catenin but neither could reduce LEF1-VP16 induced transcription (Fig. 2B,C). ΔEGF_N1 and NICD similarly regulated the Xenopus β-catenin and Tcf3-VP16 proteins (Fig. S2A,B). These results indicate that both a membrane-restricted and a nuclear form of Notch can reduce Wnt signalling and that it appears to do so at the level of β-catenin.

To confirm that Notch can modulate the expression of an endogenous Wnt target gene, we monitored the expression of *c-Myc* in HCT116 cells by quantitative PCR in the presence and absence of DAPT to disrupt Notch signalling. We first confirmed the endogenous expression level of *c-Myc* was downregulated when HCT116 cells were exposed to the Wnt inhibitor iCRT (Fig. 2D). Importantly, we found that the expression of *c-Myc* was upregulated when Notch signalling was inhibited in the presence of DAPT (Fig. 2E), indicating that Notch signalling inhibits the expression of an endogenous Wnt target gene.

### ΔEGF_N1 requires receptor trafficking to reduce β-catenin activity

**Figure 3:**
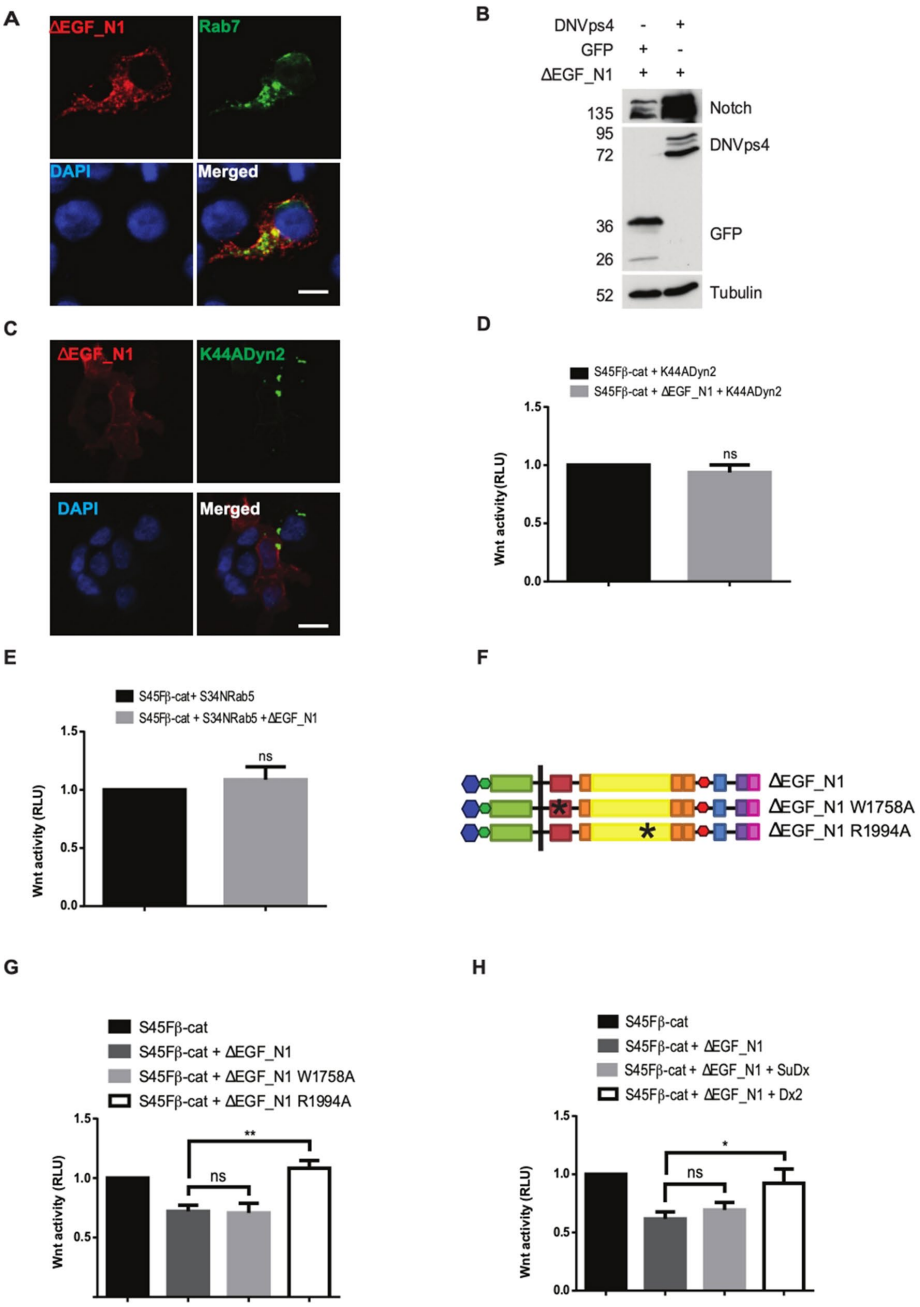
Receptor trafficking is required for ΔEGF_N1 to reduce β-catenin activity. (**A**) Membrane bound ΔEGF_N1 localised in the late endosome. Immunofluorescence analysis of ΔEGF_N1 and Rab7 proteins. Scale bar is 25 μm. (**B**) ΔEGF_N1 accumulated when receptor trafficking was blocked. Receptor trafficking was blocked by expressing a dominant negative form of Vps4. Western blot analysis of ΔEGF_N1 and DNVps4 showed more ΔEGF_N1 accumulation in cells when receptor trafficking was blocked. Expressed proteins were detected by probing the western blot with an antibody that recognises the myc epitope tag found within ΔEGF_N1 and an antibody that recognizes GFP. Tubulin is shown as a loading control. The position of molecular weight markers is shown in KDa. (**C**) ΔEGF_N1 accumulated at the cell surface when endocytosis was blocked. Immunofluorescence analysis for co-localization of ΔEGF_N1 with a DN form of Dynamin (K44ADyn2) protein. Scale bar is 25 μm. (**D**, **E**) Blocking endocytosis abrogated the ability of ΔEGF_N1 to inhibit S45Fβ-catenin-driven transcriptional activity. Endocytosis was blocked by expressing a dominant negative form of Dynamin (K44ADyn2) (**D**) or DN form of Rab5 (S34NRab5) (**E**). (**F**) Schematic showing the structure of the ΔEGF_N1, ΔEGF_N1 W1758A and ΔEGF_N1 R1994A constructs (**G**, **H**) ΔEGF_N1 requires Deltex to inhibit Wnt signalling. Luciferase assays indicated that ΔEGF_N1 R1994A (**G**) and Deltex (**H**) eliminated the ability of ΔEGF_N1 to inhibit the S45Fβ-catenin-driven transcriptional activity, while ΔEGF_N1 W1758A or Suppressor of Deltex (SuDx) had no significant effect. HEK293T cells were transfected with the Wnt reporter plasmid pTCF-AdTATA and Wnt signalling was activated by expressing S45Fβ-catenin. Experiments were performed in triplicate. pRL-CMV was used as a transfection control and cells were lysed 48 h post transfection to determine luciferase activity. Data are presented as mean fold change (± SEM) in RLU (NS *P* > 0.05; *P < 0.05, **P < 0.01, one-way ANOVA and Tukey’s post-hoc test, N = 3). Original uncropped Western Blot images of panel B are shown in Supplementary Fig. 5

Previous work has suggested that membrane localised Notch proteins can reduce Wnt signalling by interacting with β-catenin and promoting its endocytosis and degradation^15,16^. Consequently, we first looked to see whether the ΔEGF_N1 protein is found within vesicles of the endocytic pathway. We found clear association with Rab7 positive vesicles indicating that the protein is found within the late endosome (Fig. 3A). Additionally, expressing a dominant negative form of Vps4, which prevents trafficking from the late endosome to the lysosome^22^, with ΔEGF_N1 caused a marked accumulation of the protein (Fig. 3B). This suggests that the ΔEGF_N1 is trafficked through the endocytic pathway to promote its degradation. To determine whether endocytosis was important for ΔEGF_N1 mediated suppression of Wnt signalling, we co-expressed ΔEGF_N1 with dominant negative forms of Dynamin and Rab5 and monitored changes in S45Fβ-catenin induced transcription. Dynamin and Rab5 are required for vesicle scission to separate clathrin coated pits from the plasma membrane^23^ and the progression of endocytic vesicles into early endosomes^24^ respectively, and the expression of dominant negative form of Dynamin with ΔEGF_N1 restricts the ΔEGF_N1 protein to the plasma membrane (Fig. 3C). Blocking flux through the endocytic pathway by expressing either K44A-Dynamin2 or S34N-Rab5 completely abrogated the ability of ΔEGF_N1 to reduce S45Fβ-catenin transcriptional activity (Fig. 3D,E). Interestingly, S34N-Rab5 could not attenuate the effect of NICD on S45Fβ-catenin induced transcription (Fig. S3A) indicating that the expression of these proteins is not having a non-specific effect on signalling within these cells.

Deltex has been shown to induce the endocytosis of Notch to the late endosome^25^. Once within the endosome, it is the balance between Deltex and Suppressor of Deltex that controls its localisation, with Deltex causing Notch to localise to the limiting membrane^26^, whilst Suppressor of Deltex promotes its internalisation and trafficking to the lysosome^27^. To establish whether Deltex plays a role in the reduction of S45Fβ-catenin induced transcription by ΔEGF_N1, we generated a point mutation (Fig. 3F), R1994A, that disrupts its interaction with Deltex. As a control, we generated a second point mutation (Fig. 3F), W1758A, that will alter the interaction of ΔEGF_N1 with RBPj and MAML. These mutations did not alter the expression or their ability to activate an RBPj-dependent reporter gene (Fig. S3B,C). However, the R1994A mutation abolished the ability of ΔEGF_N1 to effect S45Fβ-catenin induced transcription, whilst the W1758A mutation had no effect (Fig. 3G). We also over expressed either Suppressor of Deltex or Deltex with ΔEGF_N1 to alter the balance between the two proteins. Expressing Deltex also eliminated the effect of ΔEGF_N1 on S45Fβ-catenin induced transcription, whilst expressing Supressor of Deltex had little effect (Fig. 3H). Altogether these data indicate that ΔEGF_N1 requires Deltex driven endocytosis from the plasma membrane to inhibit Wnt signalling.

### NICD does not require target gene transcription to reduce β-catenin function

**Figure 4:**
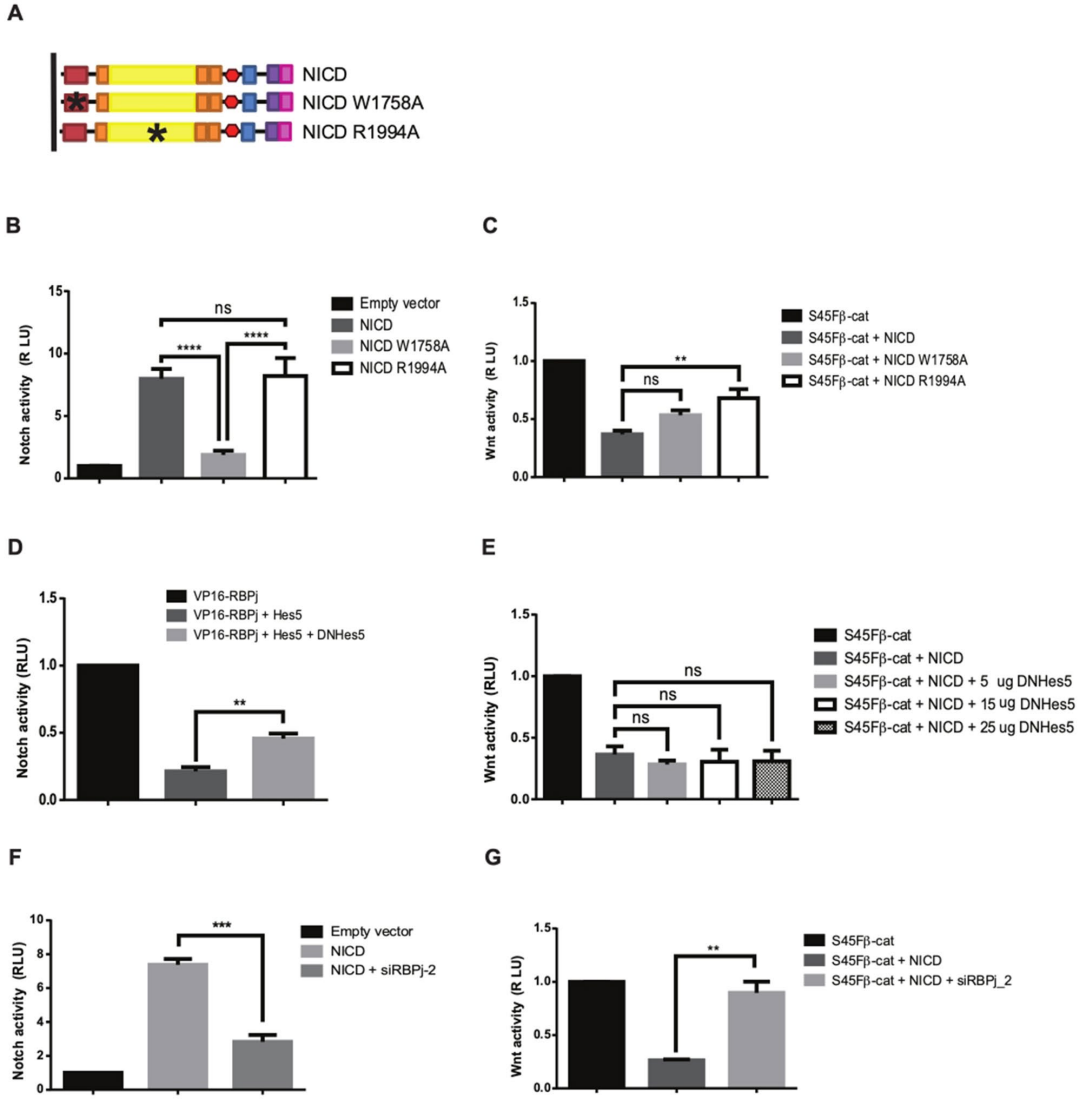
The inhibition of β-catenin-driven transcription by NICD does not require transcription of its downstream target genes. (**A**) Schematic showing the structure of the NICD, NICD W1758A and NICD R1994A constructs. (**B**) Introducing the W1758A mutation into NICD abolishes its ability to induce transcription of the Notch-dependent reporter gene p10xRbpj-luc. In contrast, the R1994A has little effect on NICD function. (**C**) Both NICD W1758A and NICD R1994A were able to reduce S45Fβ-catenin-driven transcriptional activity. (**D**) The repressor function of Hes5 is blocked by DNHes5. Expressing Hes5 with VP16-RBPj reduces the ability of VP16-RBPj to activate the pHes1-luc reporter plasmid. This inhibition of VP16-RBPj driven transcription was attenuated, as expected, by expressing a dominant negative form of Hes5. (**E**) DNHes5 is not able to eliminate the ability of NICD to inhibit S45Fβ-catenin-driven transcriptional activity. (**F**) siRNA silencing RBPj attenuates the ability of NICD to induce Notch signalling. (**G**) Inhibition of RBPj by siRNA blocked the ability of NICD to inhibit S45Fβ-catenin-driven transcriptional activity. HEK293T cells were transfected with p10xRbpj-luc or pHes1-luc (**B**,**D**,**F**) to monitor Notch signalling and pTCFAdTATA (**C**,**E**,**G**) to monitor Wnt signalling. Notch signalling was activated by expressing NICD (**B**,**F**) or VP16-RBPj (**D**). Wnt signalling was activated by expressing S45Fβ-catenin (**C**,**E**,**G**). Experiments were performed in triplicate. pRL-CMV was used as a transfection control and cells were lysed 48 h post transfection to determine luciferase activity. Data are presented as mean fold change (± SEM) in RLU (NS *P* > 0.05; **P < 0.01, ***P < 0.001, ****P < 0.0001 one-way ANOVA and Tukey’s post-hoc test, N = 3).

NICD can induce the expression of many downstream target genes, including members of the Hes and Hey family of transcriptional repressors (Fig. S4A). This raises the possibility that the inhibition of S45Fβ-catenin induced transcription by NICD simply reflects its ability to induce the expression of downstream genes. We tested this possibility in several different ways. Firstly, we generated a point mutation in NICD, W1758A (Fig. 4A), that alters the structure and function of the NICD/RBPj/MAML transcriptional activator and significantly reduced its transcriptional activity (Fig. 4B). As a control, we also generated the R1994A mutation in NICD (Fig. 4A) which did not alter its ability to induce the expression of an RBPj-dependent reporter gene (Fig. 4B). Neither mutation altered the expression of the NICD constructs, or their ability to localise to the nucleus (Fig. S4B,C). Both NICD constructs were able to reduce S45Fβ-catenin induced transcription (Fig. 4C). Secondly, we co-expressed NICD with an increasing concentration of a dominant negative form of Hes5. Although expression of the dominant negative form of Hes5 can clearly disrupt the transcriptional repressor function of Hes5 (Fig. 4D), it did not alter the ability of NICD to inhibit S45Fβ-catenin induced transcription (Fig. 4E). Similarly, the expression of the dominant negative form of Hey1 in increasing amounts did not alter the function of NICD to block S45Fβ-catenin induced transcription (Fig. S4D). Lastly, we expressed the NICD protein in cells where RBPj had been knockdown. This significantly reduced the ability of NICD to induce the expression of an RBPj-dependent reporter gene (Fig. 4F) and prevented the ability of NICD to inhibit S45Fβ-catenin induced transcription (Fig. 4G). Together these results indicate that NICD can inhibit Wnt signalling without inducing the expression of downstream target genes but requires RBPj.

### Notch interacts with β-catenin to alter its function

**Figure 5:**
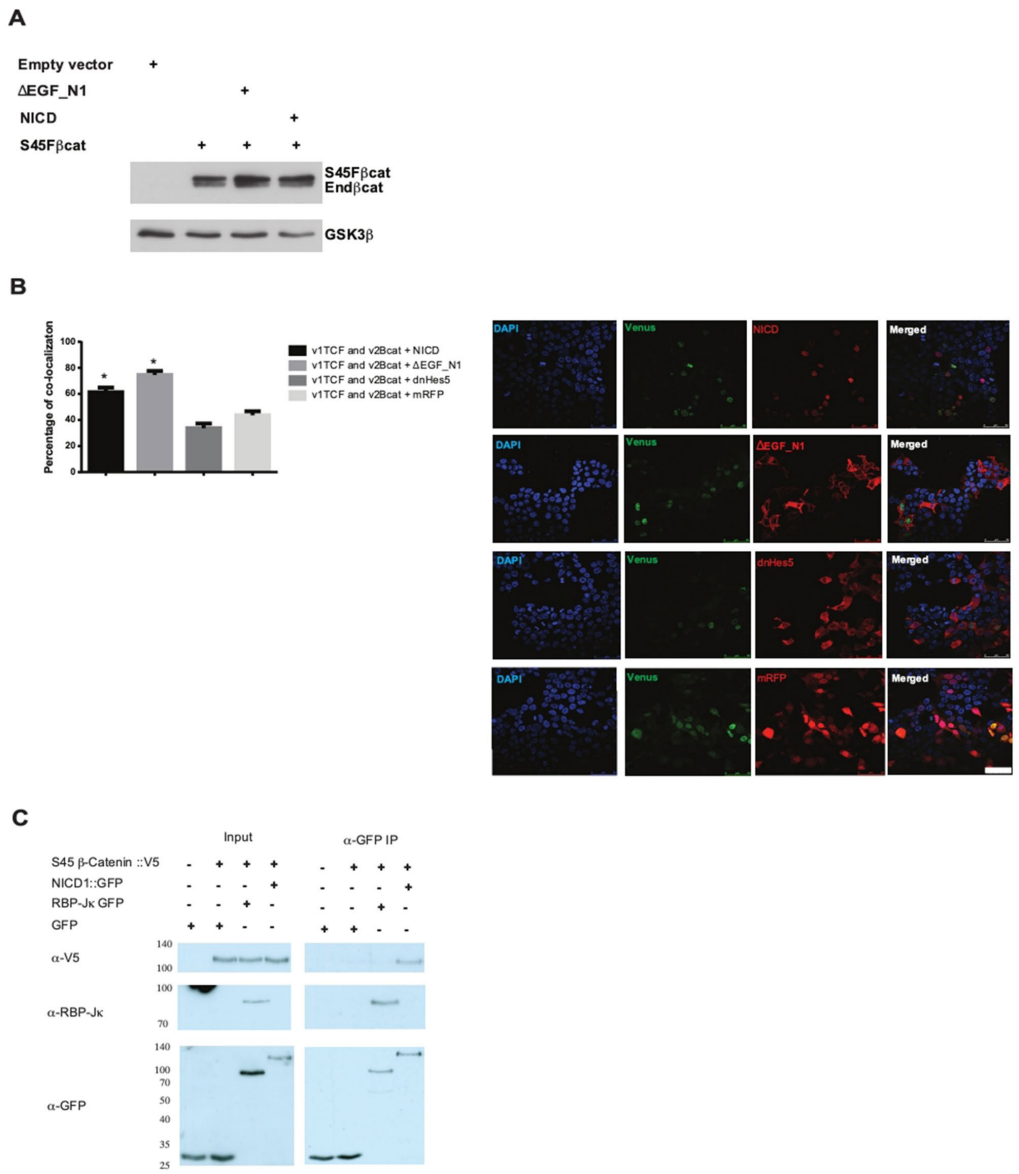
Notch inhibits β-catenin-driven transcription through an interaction between the two proteins. (**A**) NICD and ΔEGF_N1 did not alter the cytosolic levels of endogenous β-catenin or S45Fβ-catenin. Western blot analysis of cytosolic fractions of HEK293T cells expressing S45Fβ-catenin alone or with NICD or ΔEGF_N1. Note expressing S45Fβ-catenin induced the cytosolic accumulation of endogenous β-catenin. Proteins were detected with antibodies that recognise endogenous β-catenin and the myc-epitope found in the NICD and ΔEGF_N1. Glycogen synthase kinase 3 beta is shown as loading control. The position of molecular weight markers is shown in KDa. (**B**) NICD and ΔEGF_N1 did not affect the complex formation between TCFV1 and V2β-catenin. Venus fluorescence was used to monitor complex formation. RFP fluorescence was used to detect NICD, ΔEGF_N1, DNHes5 and RFP protein expression. Quantification of the number of nuclei expressing NICD, ΔEGF_N1, DNHes5 or RFP where complex formation between TCFV1 and V2β-catenin was also observed. Scale bar is 50 μm. Experiments were performed in triplicate. Data are presented as percentage co-localisation(± SEM) of NICD, ΔEGF_N1, DNHes5 or RFP with Venus fluorescence (*P < 0.05 compared to co-localisation seen with RFP one-way ANOVA and Tukey’s post-hoc test, N = 3). (**C**) Detection of a protein complex between β-catenin and NICD. GFP-tagged NICD and RBPj were isolated by immunoprecipitation from total nuclear lysates using an antibody that recognises GFP. The presence of β-catenin within these complexes was analysed by western blotting for β-catenin with an antibody that recognises the V5 epitope within the β-catenin protein. The position of molecular weight markers is shown in KDa. Original uncropped Western Blot images of panel A and C are shown in Supplementary Fig. 5.

To understand how NICD and ΔEGF-N1 inhibit S45Fβ-catenin-driven transcriptional activity, we first looked to see whether they promote the degradation of S45Fβ-catenin. As it is the soluble pool of β-catenin that is important for Wnt signalling, a cytosolic fractionation for S45Fβ-catenin, in the absence or presence of NICD and ΔEGF_N1, was performed. Expression of S45Fβ-catenin in HEK293T cells elevated the overall levels of endogenous β-catenin (lower band of the doublet, Fig. 5A) and showed accumulation of S45Fβ-catenin (upper band of the doublet, Fig. 5A) in the cytosol. Co-expression of NICD or ΔEGF-N1 had no discernible effect on the levels of endogenous β-catenin or S45Fβ-catenin.

Next, we examined the possibility that NICD and ΔEGF-N1 could prevent the formation of the β-catenin/TCF transcriptional complex. To test this hypothesis, we used a split protein complementation approach^28^, where we transfected a full length TCF4 protein fused with N-terminal half a Venus GFP (TCFV1) and a full length β-catenin protein fused with the C-terminal half of Venus GFP, (V2β catenin). Venus GFP will only refold to form a functional fluorescent protein if the TCF4 and β-catenin proteins interact^28^. We found that in the presence of NICD and ΔEGF_N1, TCFV1 and V2β-catenin still associated to form a functional Venus GFP (Fig. 5B). In fact, we found that the β-catenin/TCF transcriptional complex was more likely to form in cells expressing NICD and ΔEGF_N1 than cells expressing RFP or DNHes5 (Fig. 5B), indicating that neither Notch protein can block Wnt signalling by preventing the formation of the β-catenin/TCF transcriptional complex.

Lastly, we looked to see whether Notch could form a complex with β-catenin to alter its function. To test this, we used immunoprecipitation and we found a strong evidence for formation of a protein complex between NICD and S45Fβ-catenin (Fig. 5C). These data demonstrate that Notch forms a complex with β-catenin within the nucleus and inhibits its transcriptional activity without altering normal expression or localisation of β-catenin to the nucleus.

## Discussion

Understanding the intricacies of the interactions between the Notch and Wnt signalling pathways will help us model how cell fates decisions are controlled by these pathways during development and tissue homeostasis^29^. Furthermore, this knowledge will help us understand how these decisions go wrong during disease pathogenesis and potentially how we modulate these interactions to treat these diseases. Here, we have shown that Notch inhibits Wnt signalling in two distinct ways via membrane bound ΔEGF_N1 and nuclear NICD proteins. Firstly, endocytosis of ΔEGF_N1 sequesters β-catenin into the endocytic pathway limiting Wnt signalling. Secondly, NICD forms a complex with β-catenin within the nucleus and limits β-catenin-induced transcription. Together, this study provides a detailed mechanistic understanding of the inhibitory crosstalk between the Notch and Wnt pathways.

Previous studies have argued that Wnt signalling is inhibited by Notch either solely via a membrane restricted^14–16^ or a nuclear localised^17–19^ form of the protein. However, as many of former studies have been done with forms of the Notch protein that can be cleaved to release a nuclear localised form of the protein, it is possible to harmonise these apparently contradictory conclusions by assuming that the mechanism is through a nuclear complex. Here, we have used a form of Notch that is membrane restricted and doesn’t undergo cleavage. We have found that this form of Notch can also inhibit Wnt signalling, although less potently than NICD, and that this inhibition is dependent on the endocytosis of the protein like previous studies^14–16^. Interestingly, this inhibition of Wnt signalling by Notch is dependent on Deltex and may use the same endocytic mechanism as seen for Deltex-driven Notch signalling^30^.

In addition to the inhibition of Wnt signalling by membrane bound Notch protein, we also found clear evidence for a distinct mechanism for Wnt inhibition that occurs within the nucleus through a complex between NICD and β-catenin, consistent with previous work^14,18,19^. What hasn’t been clear previously is whether this complex directly inhibits Wnt signalling or does so via the induction of transcriptional repressors, like Hes and Hey, that then suppress the expression of Wnt target genes. Here, we show that a form of NICD that cannot induce transcription is still able to inhibit β-catenin-driven transcription. Furthermore, blocking the function of Hes and Hey by expressing a dominant negative form of the protein did not alter the ability of NICD to inhibit Wnt signalling. Together, these results argue that the effect of NICD on β-catenin-driven transcription is direct. We also find that NICD inhibits β-catenin-driven transcription more strongly than ΔEGF_N1, which may simply reflect the nuclear localisation of RBPj.

It is interesting that we have identified two distinct mechanisms by which Notch can inhibit Wnt signalling. Previous work in Drosophila on sense organ precursor and muscle progenitor cell development has suggested that Notch can suppress their development in two ways^31,32^. There is an initial repression of sense organ precursor and muscle progenitor cell development by Notch that is ligand and Suppressor of Hairless (Su(H)) independent; Su(H) is the Drosophila homologue of RBPj. This repression is then alleviated by a Wnt signal to initiate sense organ precursor and muscle progenitor cell development in a group of cells. Then there is a second ligand and Su(H) dependent Notch signal that resolves this cluster of cells to one or two cells that will complete their development into a sense organ precursor or muscle progenitor cell. Given our results, we would suggest that initial repression is mediated by a membrane bound Notch protein, whilst the second is mediated by a nuclear form of Notch. This also fits with the potency of inhibition we see. The repression mediated by the membrane bound form of Notch is modest and therefore easily alleviated by a Wnt signal to initiate development. The nuclear repression is much stronger and will overcome the Wnt signal to suppress sense organ precursor and muscle progenitor development in all but one or two cells. Interestingly, very similar observations have been made in mice during cardiac precursor cell development^15^, suggesting that this is a conserved mechanism across metazoa. These studies also show how it is important to regulate the transition from a Notch-ON/Wnt-OFF to Notch-OFF/Wnt-ON and back again during cell differentiation^2,3,33^.

## Materials and methods

### Cell culture

HCT116 cells were purchased from ATCC. Human embryonic kidney cells that stably express the large T antigen of simian virus 40 (HEK293T) were obtained from Dr Anthony Brown (Weill Medical College, Cornell University, New York, USA) and from Dr Valerie Kouskoff (Paterson Institute for Cancer Research, Manchester, UK). HCT116 and HEK293T cells were cultured in DMEM medium (Lonza, Basel, Switzerland) supplemented with 10% FBS (Biowest, Nuaillé, France) and 50 μg/ml penicillin and 50 μg/ml streptomycin (Lonza, Basel, Switzerland). Cells were maintained at 37 °C and 5% CO_2_ in a humidified incubator.

### Transfections and luciferase reporter assays

Cells were transfected using Lipofectamine and PLUS reagent (Invitrogen, Carlsbad, USA) or X-Treme transgene 9 transfection reagent (Roche, Basel, Switzerland), according to manufacturers’ instructions. HEK293T cells were plated at a density of 2 × 10^5^ cells/well of a 24 well plate. After 24 h cells were transfected in triplicate with a 250 ng DNA cocktail containing the desired DNA plasmids, 50 ng of the required reporter plasmid (p10xRbpj-luc, pTCFAdTATA or pHes1-luc), and 20 ng of pRL-CMV plasmid as an internal control. To ensure the transfections contained a constant amount of DNA, an appropriate amount of the plasmid pcDNA3.1(+) was added. The cell culture medium was changed 3 h after transfection when Lipofectamine and PLUS reagent (Invitrogen, Carlsbad, USA) were used. Cells were lysed 48 h post-transfection using 1 × Passive Lysis buffer (Promega, Madison, USA). Firefly and Renilla luciferase activities were measured using the Dual Luciferase Reporter assay system (Promega, Carlsbad, USA), according to manufacturer´s instructions, with a MicroLumatPlus plate reader (Berthold Technologies, Harpenden, UK). Data are presented as mean fold change (± SEM) in relative luciferase units (RLU), compared to β-catenin, NICD or VP16-RBPj. Statistical analysis was perform using one-way ANOVA and Tukey’s post-hoc tests using Prism software (GraphPad, La Jolla, USA) for more than two samples or with a Student *T* test for data with two samples.

### Plasmids, expression constructs and transcriptional reporters

The following plasmids were kind gifts: The **pLNCX + mWnt1** plasmid was obtained from Dr Anthony Brown (Weill Medical College, Cornell University, New York, USA). **mβ-catenin** cDNA was obtained from Geneservice (Cambridge, UK) (I.M.A.G.E. clone 5,709,247). The **pcDNA3 + mN1** plasmid was obtained from Dr Jeff Nye (Northwestern University Medical School, Chicago, USA). The **pCMX + VP16-RBPj** plasmid was obtained from Dr Tasuko Honjo (Kyoto University, Japan)^34^. The cDNA encoding **mHes5** was obtained from Dr Ryoichiro Kageyama (Kyoto University, Japan). The **pCS2 + LEF-VP16** plasmid was obtained from Dr. Rolf Kemler (Max-Planck Institute for Immunology, Freiburg, Germany). The **pcDNA3.1 Zeo + TCF4v1** and **pcDNA3.1 Zeo + v2β-catenin** plasmids were obtained from Dr Claudia Wellbrook (Faculty of Life Sciences, University of Manchester, UK). The **Xβ-catenin** plasmid was a gift from Dr Louise Howe (Weill Medical College of Cornell University, New York, USA). The plasmid **pcDNA3.1(+)**, used as an empty vector in transfections, was obtained from (Invitrogen, Carlsbad, USA). The **p10xRbpj-luc** Notch reporter plasmid was obtained from Dr Grahame MacKenzie (Lorantis, Cambridge, UK). The **pHes1-luc** reporter plasmid was obtained from Dr Ryoichiro Kageyama (Kyoto University , Japan). The **pTOPflash** Wnt reporter was obtained from Dr Louise Howe (Weill Medical College, Cornell University, New York, USA). The **pRL-CMV** reporter plasmid was obtained from (Promega, Madison, USA). The **pmRFP-C1** and **pEGFP-Rab7** plasmid were obtained from Dr Andrew Gilmore (Faculty of Life Sciences, University of Manchester, UK).

The following plasmid has already been described^35^: pcDNA3.1 myc/HisA + mβ-catenin.

The following plasmids were generated in our laboratory.

**pSecTagNC + ΔEGF_mN1:** the cDNA encoding mouse Notch 1 molecule that lacks all 36 EGF-like repeats was generated by cloning a PCR fragment (using mN1 4409F and mN1 4901R primers) digested HindIII/SacI along with a SacI/HindIII fragment of the mN1 cDNA into pSecTagNC + ΔEGF + LNR_mN1 (HindIII).

**pcDNA3 + mNICD:** the cDNA encoding myc tagged mNICD was generated by cloning the KpnI/BspEI fragment from pEGFP-N1 + mNICD into pcDNA3 + mN1 (KpnI/BspEI).

**pcDNA3.1(+) + VP16/Rbpj:** was generated by digesting pcDNA3.1(+) KpnI/EcoRI (blunted) and ligated to remove the restriction sites from HindIII to EcoRI in the pcDNA3.1(+) multiple cloning site. The VP16-Tag was cloned from pCMX-N + VP16/Rbpj (HindIII). The Rbpj cDNA was cloned from pCMX-N + VP16/Rbpj (EcoRI).

**pcDNA3.1(+) + myc-mHes5:** was generated by digesting pcDNA3.1(+) with PmeI and inserting a fragment containing the following sequences: Kozak, myc-tag epitope, EcoRI, HindIII, and BamHI, and then cloning mHes5 cDNA as an EcoRI/BamHI-digested PCR fragment generated using mHes5 73F and mHes5 663R primers.

**pcDNA3.1(+) + myc-hHey1:** was generated by digesting pcDNA3.1 (+) with PmeI and inserting a fragment containing the following sequences: Kozak, myc-tag epitope, EcoRI, HindIII, and BamHI and then cloning hHey1 cDNA (I.M.A.G.E. BC001873) as an EcoRI/BamHI digested PCR fragment generated using hHey1 98F and hHey1 11077R primers.

**pmRFP + hHes5 & pmRFP + DNhHes5:** these cDNAs were generated by digesting pcDNA3.1 + myc-hHes5 and pcDNA3.1 + myc-DNhHes5 with EcoRI/BamHI and inserting the fragment into pmRFP-C1 (EcoRI/BamHI).

**pcDNA6 V5/HisA + S45Fmβ-catenin:** was generated by excising S45Fmβ-catenin from pcDNA3.1(+)/myc-HisA + S45Fmβ-catenin (generated using site directed mutagenesis, primers mβ-cat S45F F, mβ-cat S45F R) as a KpnI/XhoI fragment and inserting it into pcDNA6/V5-HisA vector (KpnI/XhoI).

**pGL3basic + TCFAdTATA:** this Wnt reporter plasmid was generated in two steps. Initially, p10xCBF1-luc was digested with XhoI (blunted)/BglII (blunted) and re-ligated to form pGL3basic + AdTATA. pGL3basic + AdTATA was then digested with XhoI and the SalI fragment containing the 4 TCF sites from pTOPFlash was introduced.

The following plasmids were generated by mutagenesis.

**pSecTagNC + ΔEGF_mN1 W1758A and pSecTagNC + ΔEGF_mN1 R1994A:** these plasmids were generated by PCR-based mutagenesis using the QuikChange Lightning Multi Site-Directed mutagenesis kit (Stratagene, Santa Clara, USA) using pSecTagNC + ΔEGF_mN1 as a template and primers: W1758A F and R1994AmutF, respectively.

**pcDNA3 + mNICD W1758A:** this plasmid was generated by PCR-based mutagenesis using the QuikChange Site-Directed mutagenesis kit (Stratagene, Santa Clara, USA) using pcDNA3 + mNICD as a template and the primers W1758A F and W1758A R.

**pcDNA3 + mNICD R1994A:** this plasmid was generated by PCR-based mutagenesis using the QuikChange Lightning Multi Site-Directed mutagenesis kit (Stratagene, Santa Clara, USA) using pcDNA3 + mNICD as a template and the primer R1994AmutF.

**pcDNA3.1(+) + DNhHey1**, **pcDNA3.1(+) + DNmHes5:** these plasmids were generated by mutating the conserved DNA binding domain Glu-Lys-X-X-Arg (EK**R) to Alanines. This generates a non-functional protein that when transfected can dimerise with endogenous proteins disrupting their function. These DN proteins were generated by PCR-based mutagenesis using the QuikChange Lightning Multi Site-Directed mutagenesis kit (Stratagene, Santa Clara, USA) using pcDNA3.1(+) + myc-hHey1, pcDNA3.1(+) + myc-mHes5 as templates and primers: hHey1 E58AK59AR62A, and mHes5 E25AK26AR29A respectively.

For list of the PCR and sequencing primers used see supplementary table 2 (Table S2).

For list of the mutagenesis primers used see supplementary table 3 (Table S3).

### S100/P100 cytosolic fractionation

Cells were washed twice in 3 ml of 1 × TBS (10 mM Tris–HCl pH 7.4 + 140 mM NaCl) + 2 mM CaCl_2_, placed on ice and lysed in 1 ml of lysis buffer (1 × TBS + freshly added protease inhibitor cocktail set I (Calbiochem, Darmstadt, Germany)) + 10 μl of 100 mM PMSF. Subsequently, cells were poured into a cold 2 ml dounce homogenizer and sheared with 30 strokes. Cell lysates were centrifuged at 1500×*g* for 5 min at 4 °C. The supernatant was centrifuged for 90 min at 100,000×*g* in a TLA 110 rotor in a Beckman Coulter Optima TLX-120 Ultracentrifuge at 4 °C. After the spin, 100 μl of the supernatant, containing the cytosolic fraction, were mixed with 100 μl of 2 × Laemmli buffer (100 mM Tris–HCl pH 6.8, 200 mM DTT, 4% SDS, 0.2% bromophenol blue, 20% glycerol), boiled for 3 min and stored at − 20 °C.

### Western blotting

Total cell lysis and subsequent western blotting were performed as previously described^36^. For list of the primary antibodies used see supplementary table 1 (Table S1).

### Immunofluorescence

HEK293T cells were seeded on nitric acid treated coverslips at 4 × 10^5^ cells/well of a 6 well plate. Transfection was performed 24 h later, as described above. Cells were fixed 24 h post transfection for 10 min in 1 × PBS containing 4% formaldehyde. Following three washes with 1 × PBS, coverslips were incubated with α-myc-Tag rabbit primary antibody (Cell signaling, Danvers, USA) for Notch constructs, diluted 1:100 in blocking solution (3% goat serum (Biosera, Sussex, UK), 0.1% Triton-X100, and 0.05% NaN_3_ in TBS) in a humidified chamber for 1 h. Subsequently, cells were washed and coverslips were incubated with fluorescence goat α-rabbit Alexa 594 secondary antibody in blocking solution. Coverslips were mounted with VECTASHIELD mounting medium for fluorescence with DAPI H-1200 (Vector Laboratories). Images were captured with a Zeiss LSM 700, AxioObserver flexible confocal microscope (Carl Zeiss MicroImaging GmbH, Germany), using the Zeiss ZEN 2011 software (Carl Zeiss MicroImaging GmbH, Germany). The confocal software was used to determine the optimal number of Z sections when acquiring 3D optical stacks. Either maximum intensity projections of these 3D stacks or single z-section images are shown in the results. Or coverslips were mounted with Dako fluorescent mounting medium (Agilent Technologies, Santa Clara, USA). Images were collected on a Leica TCS SP5 AOBS inverted confocal using a (63 × HCX PL Apo) objective. The confocal settings were as follows, pinhole 1 airy unit, scan speed 1000 Hz unidirectional, format 512 × 512. Single z-section images where taken.

### Quantitative PCR

Total RNA was extracted from cells using peqGOLD TriFast solution (peqlab Biotechnologie GmbH, Erlangen, Germany) according to manufacturer’s instructions. A total of 2 μg of RNA were reverse transcribed to cDNA in a 20 μl reaction using the High Capacity RNA-to-cDNA Master Mix kit (Applied Biosystems by Life Technologies, Carlsbad, USA), following manufacturer’s instructions. The resulting cDNA was used as template for the quantitative PCR. Quantitative PCR was performed in triplicate, in a total volume of 20 μl, using the primers listed on supplementary table 4 (Table S4), and Fast SYBR Green PCR Master Mix (Applied Biosystems by Life Technologies, Carlsbad, USA), according to manufacturer’s instructions. The relative amounts of the PCR products were analysed using the comparative RQ method and using PPIA gene as an internal normalization control.

## Supplementary Information

Supplementary Information
