# Supplementary material for: Inhibition of Wnt signalling by Notch via two distinct mechanisms
Source: Sci Rep. 2021 Apr 27;11:9096. doi: 10.1038/s41598-021-88618-5 (PMC8079408; doi:10.1038/s41598-021-88618-5)
Supplement: Supplementary file 1 — Supplementary Information [file 41598_2021_88618_MOESM1_ESM.pdf]

Inhibition of Wnt signalling by Notch via two distinct mechanisms

Ahmet Acar[1, 2, #, *], Ana Hidalgo-Sastre[2, #], Michael K. Leverentz[2], Christopher G. Mills[2], Simon Woodcock[2], Martin Baron[2], Giovanna M. Collu[3], Keith Brennan[2, *]

Running title: Notch inhibits Wnt signalling.

Key words: Notch, Wnt, β-catenin, crosstalk, signalling

[1] Department of Biological Sciences, Middle East Technical University, Universiteler Mah. Dumlupınar Bulvarı 1, 06800 Çankaya, Ankara, Turkey

[2] University of Manchester, Faculty of Biology, Medicine and Health, University of Manchester, Oxford Road, Manchester, M13 9PT, UK

[3] Department of Developmental and Regenerative Biology, Icahn School of Medicine at Mount Sinai, One Gustave L. Levy Place, Box 1020, New York, NY 10029, USA

[#] equal contribution

*Corresponding authors: keith.brennan@manchester.ac.uk

acara@metu.edu.tr

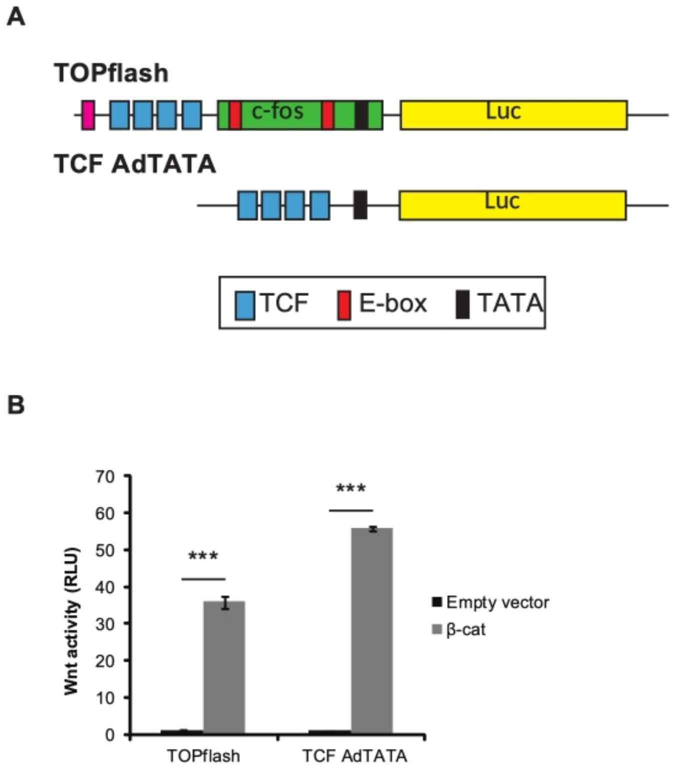

Supplementary Figure. 1 The TCFAdTATA reporter plasmid is more responsive to β-catenin-driven transcription than the TOPflash reporter plasmid and lacks E boxes. (A) Schematic showing the structure of TOPflash and TCFAdTATA reporter constructs. Note the TOPflash reporter contains E boxes that are recognised by bHLH proteins, including members of the Hes and Hey family of transcriptional repressors. **(B)** Luciferase assays were performed using these Wnt reporter plasmids. HEK293T cells were transfected with TOPflash, or TCFAdTATA to monitor Wnt signalling. Wnt signalling was activated by expressing S45Fβ-catenin. Experiments were performed in triplicate. pRL-CMV was used as a transfection control and cells were lysed 48h post transfection to determine luciferase activity. Data are presented as mean fold change (+/- SEM) in RLU (****P*<0.001 one-way ANOVA and Tukey's post-hoc test, N=3).

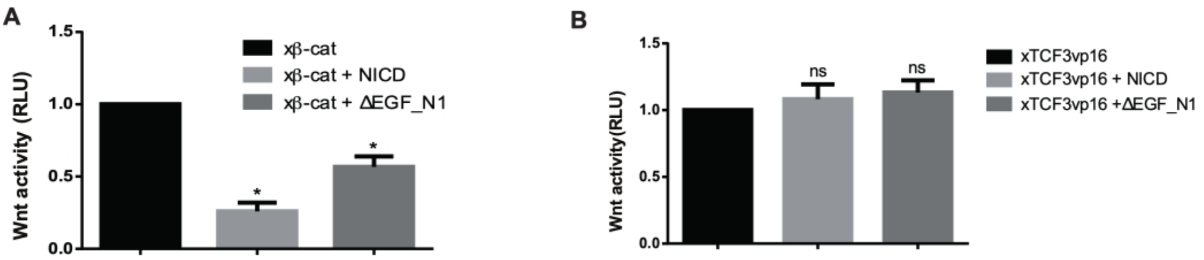

Supplementary Fig. 2 Notch-induced Wnt signalling inhibition is conserved in Xenopus. (A) ΔEGF_N1 and NICD inhibited Xenopus β-catenin (xβ-cat)-driven transcriptional activity. **(B)** ΔEGF_N1 and NICD were unable to inhibit Xenopus TCF3VP16-driven transcriptional activity. HEK293T cells were transfected with TCFAdTATA to monitor Wnt signalling. Wnt signalling was activated by expressing xβ-catenin **(A)** or xTCF3VP16 **(B)**. Experiments were performed in triplicate. pRL-CMV was used as a transfection control and cells were lysed 48h post transfection to determine luciferase activity. Data are presented as mean fold change (+/- SEM) in RLU (NS *P*>0.05 **P*<0.05 one-way ANOVA and Tukey's post-hoc test, N=3).

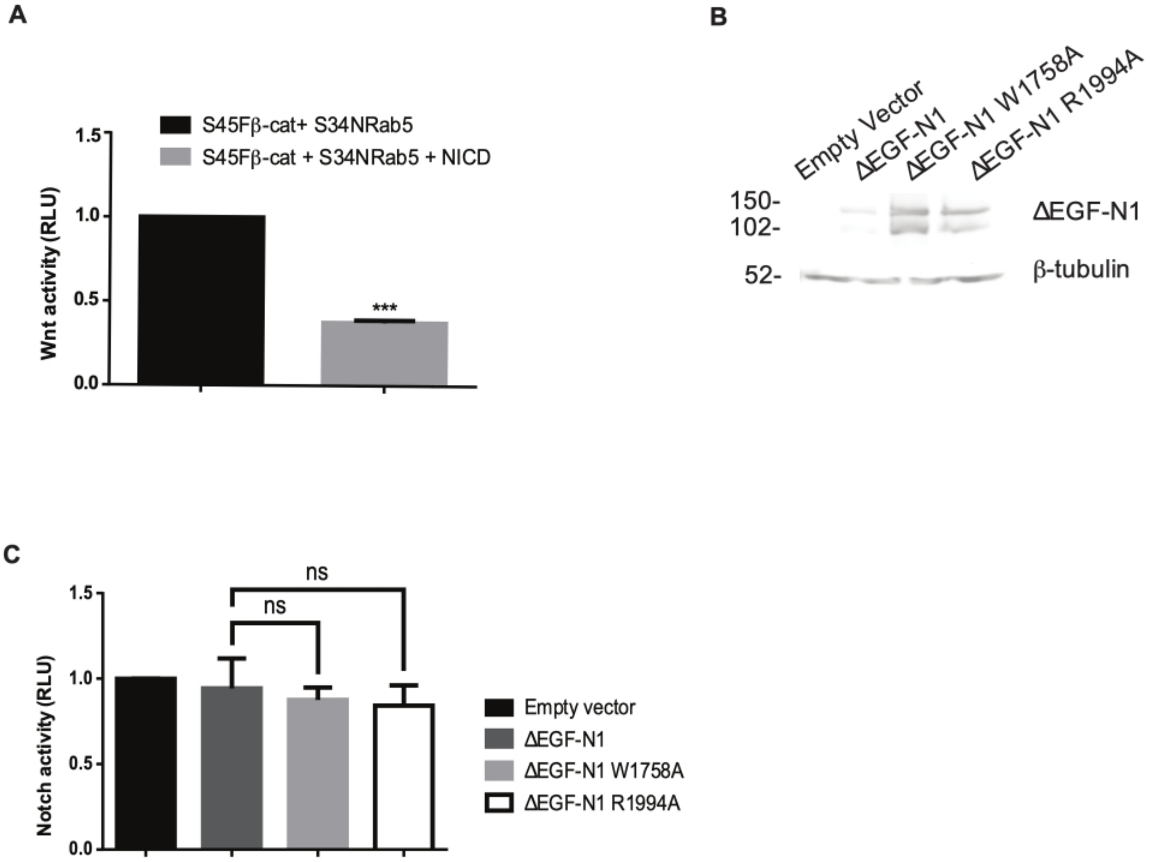

Supplementary Figure 3 Introduction of the W1758A and R1994A point mutations does not alter the expression or ability of ΔEGF_N1 to activate Notch signalling. (A) S34NRab5 couldn't attenuate the effect of NICD on S45Fβ-catenin-induced transcription. **(B)** The point mutations in the ΔEGF_N1 constructs did not alter their expression. Western blot analysis of Empty Vector, ΔEGF_N1, ΔEGF_N1, ΔEGF_N1 W1758A, ΔEGF_N1 R1994A was performed and the protein detection was achieved by probing the western blot with an antibody that recognises the myc epitope tag found within all the proteins. β-tubulin is shown as a loading control. The position of molecular weight markers is shown in kDa. **(C)** The point mutations in the ΔEGF_N1 constructs did not affect their ability to activate an RBPj-dependent Notch signalling. HEK293T cells were transfected with TCFAdTATA **(A)** or 10xRbpj-luc **(C)** to monitor Wnt and Notch signalling respectively. Wnt signalling was activated by expressing S45Fβ-catenin **(A).** Experiments were performed in triplicate. pRL-CMV was used as a transfection control and cells were lysed 48h post transfection to determine luciferase activity. Data are presented as mean fold change (+/- SEM) in RLU (NS $P>0.05$; ***$P<0.001$ one-way ANOVA and Tukey's post-hoc test, N=3). Original uncropped Western Blot images are shown in Supplementary Figure 5.

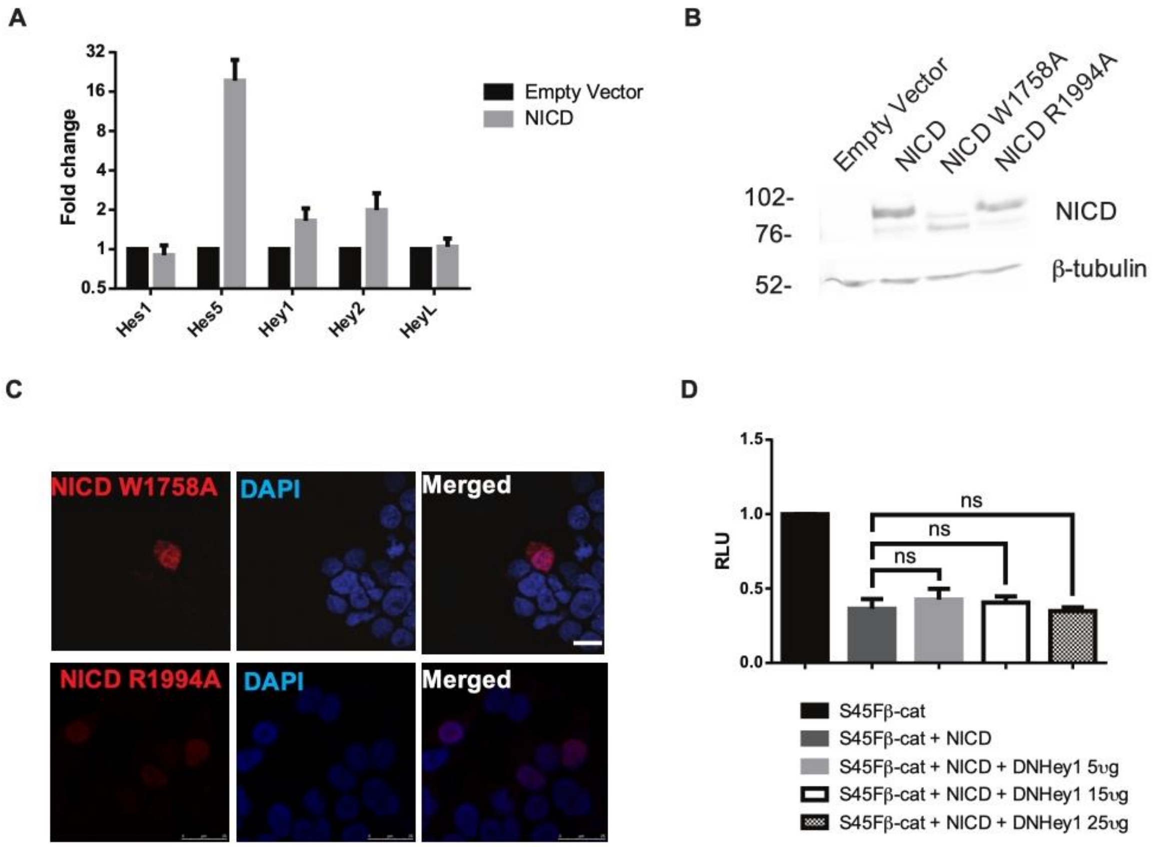

Supplementary Figure 4 Introduction of the W1758A and R1994A point mutations does not alter the expression or localisation of NICD. (A) Quantitative PCR analysis of Notch target genes. Hes5 is the most upregulated when NICD was overexpressed in HEK293T cells. **(B)** The point mutations in the NICD constructs did not alter their protein expression. Western blot analysis of Empty Vector, NICD, NICD W1758A, NICD R1994A was performed and the protein detection was achieved by probing the western blot with an antibody that recognises the myc epitope tag found within all the proteins. β-tubulin is shown as a loading control. The position of molecular weight markers is shown in kDa. **(C)** Immunofluorescence analysis of NICD W1758A and NICD R1994A proteins showed nuclear localisation like NICD. Scale bar is 25 µm. (D) DNHey1 couldn't prevent the ability of NICD to inhibit S45Fβ-catenin induced transcription. HEK293T cells were transfected with TCFAdTATA **(D)** to monitor Wnt signalling respectively. Wnt signalling was activated by expressing S45Fβ-catenin **(D)**. Experiments were performed in triplicate. pRL-CMV was used as a transfection control and cells were lysed 48h post transfection to determine luciferase activity. Data are presented as mean fold change (+/- SEM) in RLU (NS *P*>0.05; one-way ANOVA and Tukey's post-hoc test, N=3). Original uncropped Western Blot images are shown in Supplementary Figure 5.

Blots from Figure 3b

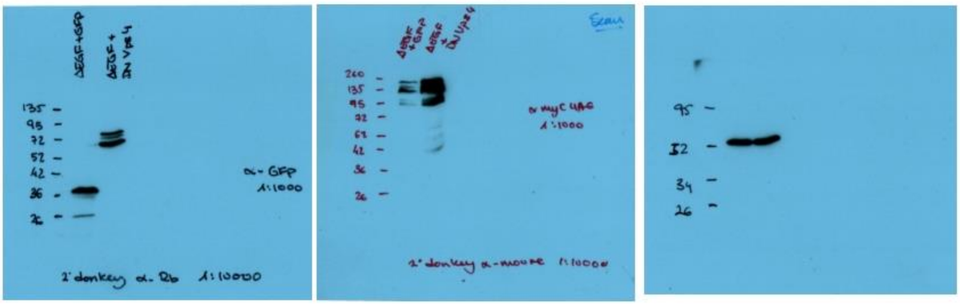

Blots from Figure 1b

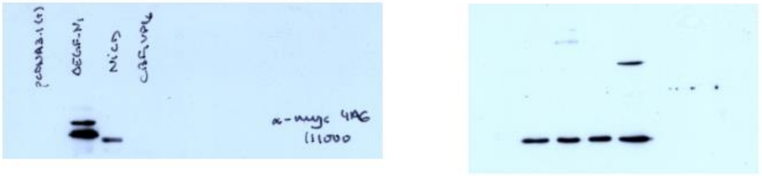

Blot from Figure 5a

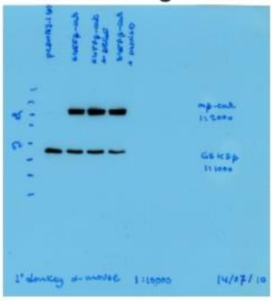

Blots from Figure 5c

S45 β-Catenin ::V5 - + + +

NICD1::GFP - - + -

RBP-Jκ GFP + + - -

GFP

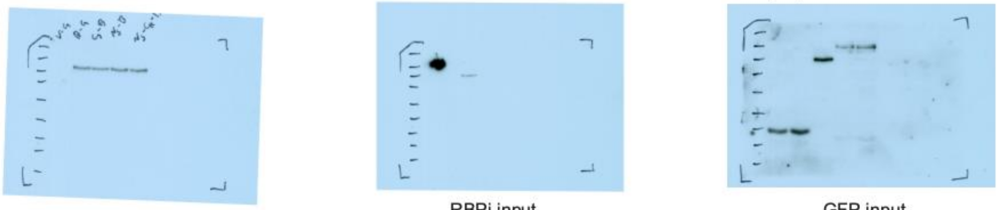

V5 input

RBPj input

GFP input

S45 β-Catenin ::V5 - + + +

NICD1::GFP - - + -

RBP-Jκ GFP + + - -

GFP

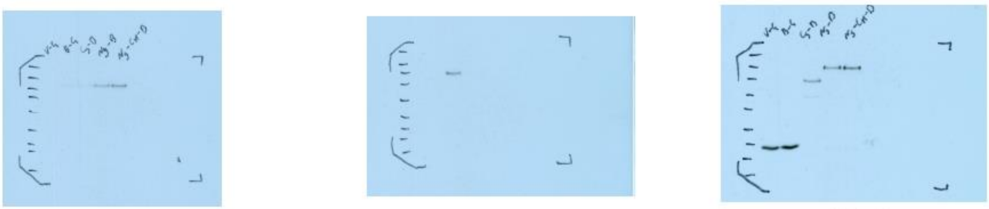

V5 IP

RBPj IP

GFP IP

Blot from Supplementary Figure 3b

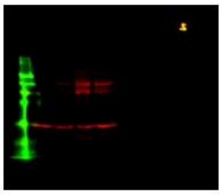

Blot from Supplementary Figure 4b

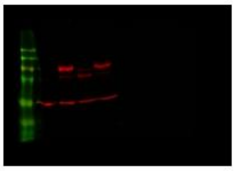

Supplementary Figure 5 Uncropped original Western Blots. Original images of full-length blots with membrane edges visible are provided. The full blot from Figure 1b is visible on the right-hand side of the photograph show where the two halves are put together. The top half has been probed for VP16 to pick up the RBPj-VP16 fusion protein and the bottom is Renilla Luciferase. The blot on the left is the top half blotted for the myc tag to detect the Notch proteins. RBPj-VP16 has not been included in figure 1b, as it hasn't been used in the manuscript.

The Supplementary information

Table S1. Antibodies used for western blot and immunofluorescence.

PRIMARY ANTIBODIES WB				
**Host species**	**Immunogen**	**Dilution**	**Supplier**	**Cat. Number**
Mouse	α-Tubulin	1:1000	Gift K.Gull Uni of Mcr	N/A
Mouse	β-catenin	1:2000	BD Transduction lab	610154
Mouse	Myc (clone 4A6)	1:1000	Upstate	05-724
Mouse	Renilla luciferase	1:500	Cell Signaling	2272
Rabbit	RFP	1:1000	MBI	PM005
Mouse	V5	1:1000	Invitrogen	R960-25
Mouse	VP16	1:200	Santa Cruz	sc-7545
**PRIMARY ANTIBODIES IF**				
Rabbit	Myc-Tag	1:400	Cell signaling	2272
**SECONDARY ANTIBODIES WB**				
Donkey	Mouse IgG	1:10000	Jackson	715-035-150
Donkey	Rabbit IgG	1:10000	Jackson	715-035-152
**SECONDARY ANTIBODY IF**				
Goat	Rabbit Alexa 594	1:400	Molecular Probes	A11037

Table S2. PCR and sequencing primers

Primer name	Sequence (5'-3')
mN1 4409F	GGTAAAGCTTCAGATTGAGGAGGCATGTGAG
mN1 4901R	GCTTGAAGACCACGTTGGTGT
mN1 5042F	TAGTAAGCTTGAGCTGGACCCTATGGACAT
mN1 5589R	TGCTGCTGAGTCCACTGTCT
mHes5 73F	TAGCGAATTCTGGCATGGCACCTAGTACCGTGG
mHes5 663R	TCGTGGATCCTGAACTGCGGCTGGGGAATGTC
hHey1 98 F	TAGCGAATTCTATGAAGCGTGCTCACCCCGAGT
hHey1 1077 R	TAGCGGATCCTCTTAGCAACAGTCCAGCCCA

Table S3. Mutagenesis primers.

Primer name	Sequence (5'-3')
mβ-cat S45FF	ACCACCACAGCTCCTTTCCTGAGTGGCAAGGGC
mβ-cat S45FR	GCCGTTGCCACTCAGGAAAGGAGCTGTGGTGGT
W1758A F	CAGCATGGCCAGCTC**GCG**TTGCCTGAGGGTTTG
W1758A R	GAAACCCTCAGGGAA**CGC**GAGCTGGCCATGCTG
R1994AmutF	TTGCATTGGGCGGCC**GTG**GTGAACAATGTGGAT
hHey1E58AK59AR62A	GGAGAGGAATAATT**GCGGCG**CGCCGA**GCA**GACCGGATCA ATAAC
mHes5E25AK26AR29A	GGAAGCCGGTGGTG**GCGGCG**ATGCGT**GCG**GACCGCATCA ACAGC

Table S4. Quantitative PCR primers.

Primer name	Sequence (5'-3')
hHey1-real5'	GGCAGGAGGGAAAGGTTACT
hHey1-real3'	GCTGGGAAGCGTAGTTGTTG
hHey2-real5'	AAGATGCTTCAGGCAACAGG
hHey2-real3'	CGCAACTTCTGTTAGGCACTC
hHes1-real5'	TCTGAGCCAGCTGAAAACAC
hHes1-real3'	CTCGGTACTTCCCCAGCAC
hHes5-real5'-new	CCCAAAGAGAAAAACCGACTG
hHes5-real3'-new	GCTTGGAGTTGGGCTGGT
hHeyL-real5'	AGACCGCATCAACAGTAGCC
hHeyL-real3'	CAAAGAATCCTGTCCCACCA
hPPIA-real5'	ATGCTGGACCCAACACAAA
hPPIA-real3'	TTTCACTTTGCCAAACACCA
